# Diet and exercise knowledge and practices for diabetes care within families in Senwabarwana

**DOI:** 10.4102/safp.v66i1.5767

**Published:** 2024-01-30

**Authors:** Mabitsela H. Mphasha, Linda Skaal, Tebogo Mothibal

**Affiliations:** 1 Department of Public Health, Faculty of Healthcare Sciences, University of Limpopo, Polokwane, South Africa; 2 Department of Research, Faculty of Healthcare Sciences, Durban University of Technology, Durban, South Africa; 3 Faculty of Healthcare Sciences, University of Limpopo, Polokwane, South Africa

**Keywords:** family members, patient care, knowledge, diet, exercise

## Abstract

**Background:**

Family members (FMs) are a valuable source of support, as the bulk of daily diabetes treatment occurs at home. Family members’ insufficient understanding of patient support can lead to poor diabetes outcomes. Lack of knowledge about good diet and exercise can lead to unhealthy food preparation and sedentary lifestyles, affecting patients and increasing the risk of diabetes. This study aims to fill the gap in the level of knowledge of FMs relating to appropriate care for diabetic patients under their care. This will relate specifically to diet and exercise.

**Methods:**

A cross-sectional survey conducted in Senwabarwana, Limpopo province, involved 200 FMs caring for diabetic patients for at least 6 months. Their experience could offer valuable insights into the competence of their care. Data were collected regarding knowledge and practice using a close-ended questionnaire, with Likert scale responses and SPSS analysis, including descriptive statistics and chi-squared tests. Knowledge was assessed on a scale ranging from poor to excellent: poor (0% – 50%), fair (51% – 60%), good (61% – 74%) and excellent (> 75%). Practice was assessed as poor (0% – 50%), fair (51% – 69%) and good (70% – 100%).

**Results:**

Thirty-one percent of participants demonstrated excellent knowledge and only 9% demonstrated good practice. Unfortunately, 53% stated that obese patients with diabetes should skip meals to lose weight. Only 3.5% and 19%, respectively, are familiar with recommendations for exercise and glucose monitoring. Barely 35.5% of FMs eat breakfast every day, while 87.5% report exercising.

**Conclusion:**

Few FMs possess excellent diabetes management knowledge but still indulge in bad practices, increasing their risk of developing diabetes. Additionally, they may potentially cause health problems for patients.

**Contribution:**

Family-centred behaviour change intervention is recommended.

## Introduction and background

Diabetes mellitus (DM) affects 537 million individuals between the ages of 20 and 79 worldwide, with 24 million adults affected in Africa.^1^ Furthermore, 13 million Africans are living with undiagnosed diabetes.^1^ South Africa’s diabetes prevalence has significantly increased from 4.5% in 2010 to 12.7% in 2019.^2^ In Limpopo province, diabetes prevalence is 9.94%, while in rural areas of the province such as Senwabarwana it is 10.44%.^3^ The observed rise in diabetes prevalence can be attributed to poor diet, physical inactivity and other lifestyle behaviours such as alcohol use and smoking. Moreover, environment, social pressures (social cohesion, social capital and social support) and economic factors may influence the primary behaviours – diet and physical inactivity. Elements related to the availability of food (food insecurity and access),^4^ healthcare (affordability, accessibility and quality) are also believed to affect the management of diabetes.^5^ Proper nutrition and regular exercise play crucial roles in diabetes management.^1^ Family members (FMs) of patients with diabetes are already susceptible to the disease.^6^ Diabetes, primarily driven by unhealthy dietary habits and a lack of physical activity, is strongly associated with obesity, a major risk factor.^7^ Insufficient knowledge about making healthy lifestyle choices is a significant contributor to obesity.

Knowledge of FMs relating to importance of diet and physical activity plays a pivotal role in healthcare,^8,9^ as it contributes to improving the quality of life of patients under their care and also helps prevent associated complications. Moreover, it may even reduce the risk of developing diabetes in those who are vulnerable.^10^ In order to improve knowledge of FMs, home visits, community awareness campaigns and joint family–patient consultations should be promoted. Nevertheless, it is important to note that, in the context of a chronic care model, knowledge should be integrated with other components. Isolated components of care do not appear to be sufficient for achieving improved clinical outcomes.^11^ However, a lower level of knowledge is associated with a higher risk of diabetes.^12^ According to a Saudi Arabian study, attempts to increase public awareness and empowerment on diabetes must be integrated into current healthcare systems and practices.^13^ Diabetes knowledge is insufficient worldwide, therefore, increasing public awareness of diabetes including reduction in behaviours associated with causing diabetes, for example, ultra-processed or refined foods with excess fats and lack of exercise. Therefore, these will consequently reduce the likelihood of developing the disease.^11^ Adopting good behaviour is not guaranteed by knowledge alone, but bad behaviour is largely associated with lack of knowledge.^14^ Additionally, integrating knowledge with a desire to change one’s conduct produces positive behavioural change.^14^ Adequate knowledge and positive behaviour are closely linked.

Engaging in appropriate diabetes management or preventative practices has shown to affect quality of life, self and overall well-being of patients,^15^ including maintaining better glycaemic levels. Physical inactivity, high calorie intake and consuming fatty, sugary and sweet meals were all found to have a substantial impact on diabetes.^16^ Health is the capacity to adapt and maintain composure in the face of social, physical and emotional issues.^17^ The management of type 2 diabetes and the prevention of the illness depend heavily on behavioural modifications and comprehensive lifestyle interventions.^18^ Positive family health practices are essential because FMs care for patients at home and influence the patient’s diabetic treatment.

Family members are a vital source of support in the daily management of diabetes.^19^ Despite their essential role, there is a significant gap in information regarding the knowledge and practices of FMs of individuals living with diabetes. This study aims to address this gap, recognising the critical need for family support in diabetes care. Family support has been linked to improved diabetes outcomes, enhanced quality of life, better health status and reduced risk of diabetic complications.^20^ Knowledge and practices of FMs in diabetes care can directly impact the health and well-being of patients. Evaluating their understanding and behaviours related to diet and exercise is crucial for enhancing patient care. Family members serve as a vital support system for individuals with diabetes. When equipped with the right knowledge and practices, they can significantly contribute to managing the condition, relieving the burden on healthcare facilities and enhancing the overall quality of life for patients.^20^ This can be done by offering emotional support, assisting with medication administration, promoting a healthy lifestyle and helping with routine blood sugar monitoring, FMs play a critical role in supporting diabetic patients. Additionally, they can play a crucial role in identifying and managing diabetic emergencies. There is variability in the efficacy of FMs’ assistance for individuals with diabetes.^19^ It is dependent upon their familiarity with managing diabetes, their ability to communicate with healthcare professionals and the patient’s readiness to receive assistance. Enhancement of this support’s efficacy can be achieved through education and transparent communication within the family. Moreover, FMs should adopt healthier lifestyle choices for the entire family, as this can benefit both the patient and at-risk FMs.

Furthermore, FMs of patients face an increased risk of developing diabetes themselves because of shared genetic and lifestyle factors.^4^ Assessing their knowledge and practices can help identify areas where preventive measures can be implemented to reduce the risk of diabetes within the family. It is important to consider the cultural context of the Senwabarwana region, deeply rooted in Sepedi culture and the principles of *botho (ubuntu),* which emphasise compassion and mutual care. This cultural backdrop underscores the significance of involving FMs in the study, as they often play a central role in healthcare. This study aims to assess the knowledge and practices related to diet and exercise for diabetes care within families in Senwabarwana, Limpopo province.

## Methodology

### Research method and design

A cross-sectional survey was used in this investigation. Cross-sectional surveys provide a snapshot of diet and exercise knowledge at a specific point in time. This is valuable for assessing the current state of knowledge and practices among families in Senwabarwana regarding diabetes care.

### Setting

The study was conducted in the clinics of Blouberg municipality, Senwabarwana Region, Capricorn district, Limpopo province, South Africa. The clinics educate patients on diabetic nutrition and exercise so that they can make changes in their daily routines at home with the support of FMs.

### Study’s population and sample

In this context, family refers to the individuals living with diabetic patients in the same household. Family members who were not currently undergoing diabetes treatment were eligible for consideration for participation, following an inquiry about their current treatment status. The sample size was established using data from all 406 diabetic patients enrolled as treatment receivers in the clinics of the Blouberg municipality. Family members of patients living with diabetes were chosen using simple random sampling. Family members were given the numbers, and then a random selection was made from them. Sample size was calculated using Yamane’s formula,^21^ which is:


$$n=N÷1+N{\left(e\right)}^{2},$$ [Eqn 1]

where *n* = Sample size, *N* = Population size (*N* = 406) and *e* = error margin (5%). The sample was rounded up to 200 from a total of 203.

Only FMs of patients who have been caring for patients for at least 6 months were included, because they are likely to have a deeper understanding of the challenges and complexities involved in diabetes care, and their experience can provide valuable insights into the nuances of diet and exercise knowledge and practices. Restricting the study to FMs who are older than 18 ensures that participants are legally capable of providing informed consent.

### Data collection

The questionnaire used in this study consisted of three sections: demographics, knowledge and practices. There were a total of 22 questions related to knowledge, with 11 focusing on diet and 11 on exercise. Additionally, there were 15 questions in total for practices, with 11 concerning dietary practices and four related to exercise practices. The content for the knowledge and practice sections of the questionnaire was adapted from similar tools used in previous studies by Muchiri, Rheeder and Gericke,^22^ and Le Roux et al.^23^

To ensure reliability, the questionnaire underwent modification and a pilot study, the results of which did not demonstrate any need to modify the final questionnaire. Content and face validity were ensured through the input of dieticians and supervisors who assessed the questionnaire’s relevance. The questionnaire was self-administered in the participants’ native language and a competent bilingual translator was employed to accurately and culturally sensitively translate the questionnaire from English to Sepedi. This translation maintained the accuracy and clarity of the questions while adapting them culturally. To verify the accuracy and consistency of the Sepedi version, back translation was performed by an independent bilingual translator in comparison to the original English questionnaire.

### Data analysis

The data underwent analysis using Statistical Package for Social Sciences, version 27. Descriptive statistics were employed to compute frequency distributions. Relationships were assessed with a 95% confidence level through the chi-squared test, where a significance level of 0.05 (*p* < 0.05) was used to determine statistical significance. Knowledge and practice were evaluated based on frequency scores, distinguishing between correct and incorrect responses. For knowledge assessment in this study, a scale ranging from poor to excellent was utilised. Poor knowledge was defined as a total score of 0% – 50%, fair knowledge encompassed scores between 51% and 60%, good knowledge ranged from 61% to 74% and excellent knowledge was indicated by scores exceeding 75%. Practice was assessed on a general scale with a maximum score of 100%. It was categorised as poor, fair or good practice. Poor practice was indicated by a total score between 0% and 50%, fair practice included scores between 51% and 69% and good practice was associated with scores falling between 70% and 100%.

### Consideration of ethical issues

This research project was approved by, a committee responsible for assessing and issuing ethical certificates at the University of Limpopo. This manuscript derives from the corresponding authors’ thesis for PhD in Public Health; this work was included with an ethical approval number. Limpopo Department of Health granted approval. Individual informed consent process was used, and participants signed a consent form indicating voluntary participation. Participants were made aware that leaving the research at any time without repercussions was completely their choice. The identities of the participants were kept a secret and confidential.

### Ethical considerations

This study is part of bigger study approved by Turfloop Research and Ethics Committee (TREC/35/2019: PG) and to access patients in the clinics was given by the Limpopo Department of Health with ref: LP 201903-007. All participants provided written informed consent. Participation was voluntary and participants were informed about their rights to withdraw from the study at any stage without penalty. Privacy and confidentiality of the participants’ data were also maintained.

## Results

### Demographic profile of participants

According to Table 1, most participants (73%) were younger than 60 years, females (76%), had at least a secondary education (in some cases a higher education) and 54% were unmarried. Only 35% of participants made more than R1000.00 per month, and 89% of participants had additional FMs who earned money. The majority of participants (78%), who lived with two to six FMs, had at least one earning FM. Most never accompanied diabetic FMs to appointments with dieticians (93.5%) and physiotherapists (96.5%) in the previous 6 months.

**TABLE 1:** Demographic profile of participants (*n* = 200).

Demographic data	*n*	%
**Age groups**		
≤ 60 years	146	73.0
> 61 years	54	27.0
**Gender**		
Male	48	24.0
Female	152	76.0
**Education**		
Primary or no schooling	72	36.0
Secondary and tertiary	128	64.0
**Marital status**		
Single	108	54.0
Married	92	46.0
**Number of family members at home**		
2–6	156	78.0
7–12	44	22.0
**Income**		
No income	67	33.5
≤ R1000.00	63	31.5
> R1000.00	70	35.0
**Number of family members with income**		
None	23	11.5
1–2	170	78.5
3–4	30	10.0
**Did you go with a family member who has diabetes to a dietician appointment within the previous 6 months?**		
Yes	13	6.5
No	187	93.5
**Did you go with a family member who has diabetes to a physiotherapist appointment within the previous 6 months?**		
Yes	7	3.5
No	193	96.5

**FIGURE 1:**
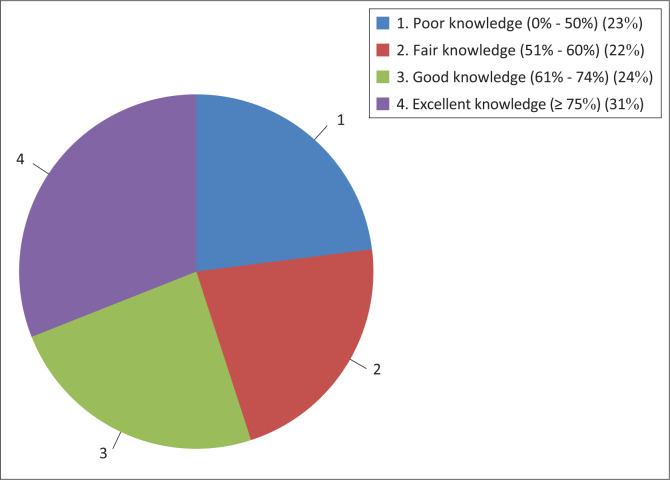
Participants’ general knowledge of nutrition and diabetes care.

Figure 1 demonstrates that 31% of individuals had excellent knowledge, followed by 24% who had good knowledge and 22% who had fair knowledge.

Table 3 shows that 53% of participants think that diabetics who are overweight should skip meals to reduce weight.

Table 2 shows that knowledge levels are not substantially associated with age (*p* = 0.998), education (*p* = 0.692), marriage (*p* = 0.114) or income (*p* = 0.762). Levels of knowledge do, however, significantly correlate with gender (*p* = 0.017).

**TABLE 2:** General family knowledge by socio-economic status.

Knowledge according to socio-demographic characteristics	Overall knowledge				χ^2^	*p*
	Poor knowledge	Fair knowledge	Good knowledge	Excellent knowledge		
**Age**						
≤ 50 years	26	26	28	35	0.044	0.998
> 50 years	19	19	20	27		
**Gender**						
Male	14	5	17	11	10.227†	0.017
Female	31	40	31	51		
**Education**						
Primary or no schooling	19	17	15	22	1.460	0.692
Secondary and tertiary	25	29	33	40		
**Marriage**						
Single	29	22	29	27	5.942	0.114
Married	16	23	19	35		
**Income**						
No income	10	9	10	15	1.211	0.762
≤ R1000.00	12	19	30	32		
> R1000.00	14	18	13	18		

† , Indicates statistical significance at 95% confidence interval.

**TABLE 3:** Diet-related knowledge in diabetes care (*n* = 200).

Diet-related knowledge	Yes		Not sure		No	
	*n*	%	*n*	%	*n*	%
Nutrition is essential in diabetic management.	181	90.5	1	0.5	18	9.0
Consuming fruits and vegetables is necessary as most of them lower blood glucose levels.	177	88.5	4	2.0	19	9.5
To control blood sugar, patients with diabetes should consume small, frequent meals on a regular basis.	162	81.0	4	2.0	34	17.0
For people with diabetes, whole grains that are high in fibre are suggested as a nutritious source of carbohydrates.	153	76.5	5	2.5	42	21.0
Overweight patients with diabetes are advised to skip meals in order to lose weight.	106	53.0	10	5.0	84	42.0
It is advised to trim visible fat from red meat and skinless chicken when preparing meat for patient.	128	64.0	2	1.0	70	35.0
A patient with diabetes may experience higher blood glucose levels if they consume large portions of food all at once.	129	64.5	3	1.5	68	34.0
Are high-fat dairy products and foods rich in animal protein recommended in the diet?	118	59.0	5	2.5	77	38.5
Diabetes patients should limit their intake of salty foods, including processed foods that are high in sodium.	140	70.0	4	2.0	56	28.0
Consumption of sugary foods, especially avoiding added sugar in food and beverages, should be reduced.	143	71.5	6	3.0	51	25.5
Fried foods and other high-fat foods should be avoided.	147	73.5	3	1.5	50	25.0

Table 4 shows that most participants (89.5%) are unaware of the recommended amount of exercise or the 75.5% acceptable blood glucose level.

**TABLE 4:** Exercise knowledge in diabetes care (*n* = 200).

Exercise-related knowledge	Yes		Not sure		No	
	*n*	%	*n*	%	*n*	%
Exercise is crucial for managing diabetes and lowers blood sugar levels.	170	85.0	3	1.5	27	13.5
Exercise helps diabetics maintain healthy blood glucose, blood pressure and cholesterol levels.	167	83.5	2	1.0	31	15.5
Patients with diabetes who exercise can avoid or lessen health issues or complications caused by their diabetes.	162	81.0	6	3.0	32	16.0
If a diabetic person has pain, shortness of breath, or dizziness when exercising, it is advised that they stop.	148	74.0	5	2.5	47	23.5
When a diabetic patient exercises for an extended period of time, it is advised that they eat before and after, as well as consume fast-acting carbohydrate beverages while exercising.	151	75.5	3	1.5	46	23.0
It is recommended for patients to monitor their blood sugar levels before and after physical activity	150	75.0	4	2.0	46	23.0
When patient’s blood glucose is high (above 13.9 mol/L) right before activity, they should wait until their blood glucose returns to normal to avoid ketoacidosis.	135	67.5	10	5.0	55	27.5
A patient is advised to exercise at least 3 days a week for 30 min at the very least.	7	3.5	14	7.0	179	89.5
When exercising while on insulin and having diabetes, the patient should not inject oneself on the thighs.	6	3.0	10	5.0	184	92.0
Exercise causes the body’s level of insulin to drop.	48	24.0	13	6.5	139	69.5
Patients should have blood glucose levels between 4 mmol/L and 8 mmol/L.	38	19.0	11	5.5	151	75.5

Significant correlation between practice levels and age is shown in Table 5 (*p* = 0.025). Additionally there is no correlation between gender (*p* = 0.405), education (*p* = 0.715), marriage (*p* = 0.891) and income (*p* = 0.772).

**TABLE 5:** Cross tabulation: Overall practice of family members by socio-demographic profile.

Practice vs. socio-demographic profile	Practice			χ^2^	*p*
	Bad practice	Fair practice	Good practice		
**Age**					
≤ 50 years	53	57	5	7.389†	0.025
> 50 years	32	40	13		
**Gender**					
Male	22	23	2	1.808	0.405
Female	63	74	16		
**Education**					
Primary or less	31	33	8	0.671	0.715
Secondary or more	54	63	10		
**Marriage**					
Single	47	51	9	0.232	0.891
Married	38	46	9		
**Income**					
No income	13	16	13	0.533	0.772
≤ R1000.00	20	32	25		
> R1000.00	34	28	19		

† , Indicates statistical significance at 95% confidence interval.

Table 6 reveals that only 35.5% of the individuals (or more than one third) frequently have breakfast in addition to meat, poultry, fish, mopani worms, eggs and milk (29%).

**TABLE 6:** Nutritional practices adopted by family members engaged in diabetes care (*n* = 200).

Nutrition related practices	Regularly (4 or more times a week)		Sometimes (1–3 times a week)		Never	
	*n*	%	*n*	%	*n*	%
Eating of breakfast in the previous week.	71	35.5	119	59.5	10	5.0
How frequently did you eat dinner and lunch the previous week?	138	69.0	62	31.0	0	0.0
Consumption of fried and fatty foods.	16	8.0	127	63.5	57	28.5
Intake of sugary food and drinks.	10	5.0	129	64.5	61	30.5
At what frequency do you drink alcoholic beverages?	2	1.0	50	25.0	148	74.0
Consumption of vegetables.	36	18.0	155	77.5	9	4.5
Intake of fruits.	34	17.0	160	80.0	6	3.0
How frequently do you consume milk, beef, poultry, fish, mopani worms and eggs?	58	29.0	136	68.0	6	3.0
How frequently do you eat high-fibre meals like whole grains, beans, lentils and other legumes?	188	94.0	12	6.0	0	0.0
How often do you eat carbohydrates like bread, rice, cereal and samp?	17	8.5	120	60.0	63	31.5

Table 7 demonstrates that 87% of individuals engage in physical activity. In the past month, only 32% of people worked out for at least 30 min three times a week.

**TABLE 7:** Exercise practices of family members caring for patients with diabetes (*n* = 200).

Participants’ behaviour in support of patients with diabetes	Yes		No	
	*n*	%	*n*	%
Do you engage in physical activity?	174	87	26	13
Along with home chores like laundry and cleaning, did you spend at least 30 min, three times a week, stretching or doing muscle-strengthening exercises like lifting weights?	78	39	122	61
Do any of the following exercises count toward your three required minimum-time activities each week: walking, jogging, cycling, climbing stairs, or any other aerobic activity?	77	38.5	123	61.5
Have you worked out for at least 30 min on 3 days last week?	64	32	136	68

**FIGURE 2:**
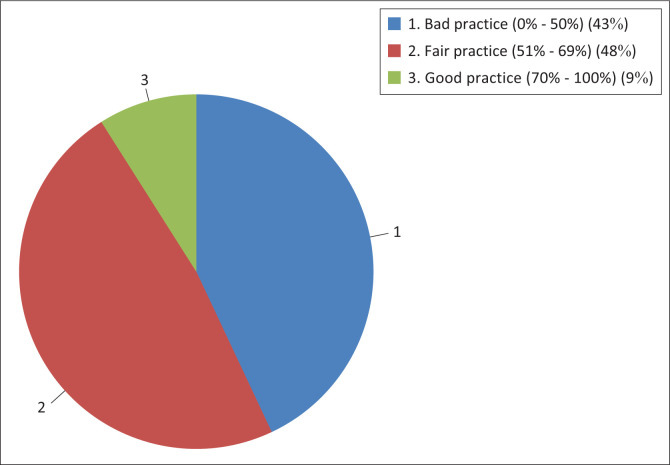
Participants’ general practice.

Figure 2 shows that only 9% participants had good (70% – 100%), fair (51% – 69%) (48%) and 43% had bad (0% – 50%) overall nutrition and exercise practice.

## Discussion

This study focused on assessing the knowledge and practices of patient FMs in managing diabetes through exercise and diet, recognising the pivotal role FMs play in diabetes care. Results revealed that only 31% of the participants exhibited excellent knowledge of diabetes management through exercise and diet. This finding aligns with an Ethiopian study that highlighted the lack of awareness about diabetes among non-diabetic community members.^11^ Family members possessing substantial knowledge about diabetes and its potential consequences can play a critical role in supporting FMs with the condition. They can assist in ensuring that patients adhere to medical appointments and lifestyle modifications necessary for diabetes management.^24^ However, the study showed a need for knowledge empowerment to enhance effective support to patients in achieving improved diabetes outcomes. The study recommends an urgent need of raising awareness about proper nutrition and exercise in the context of diabetes treatment. The recommended awareness campaign should aim to educate individuals about the essential do’s and don’ts in managing diabetes effectively while also safeguarding patients’ well-being. Given that FMs are already at risk because of shared genetics and lifestyle factors, empowering them with knowledge can initiate lifestyle adjustments to reduce or prevent the onset of diabetes.^25^ Providing adequate information can inspire and motivate people to adopt healthier lifestyles.^14^ The participants in this survey demonstrated a lack of awareness regarding the potential hazards of consuming large portions, the importance of avoiding high-fat dairy products, and the misconception that obese patients should skip meals to lose weight. This finding aligns with a Kenyan study that indicated non-diabetic FMs of patients had insufficient knowledge of diabetes diets.^26^

Consumption of high-portioned, fatty meals has been linked to the development of obesity.^27,28^ Notably, obesity not only increases the risk of diabetes among FMs because of shared genetics and lifestyle but also significantly impacts the incidence and consequences of diabetes among patients themselves.^7^ Additionally, the survey revealed that more than two-thirds (63%) of adults reported consuming fatty, sugary and salty foods one to three times per week, while less than one-third (35%) reported having breakfast daily. This contradicts the assumption that skipping meals is primarily because of budgetary constraints, as a significant majority (69%) reported regularly consuming both lunch and dinner. This pattern of increased fat and oil consumption is observed not only in South Africa but also in other emerging nations.^29^ Furthermore, the survey found that only 38.5% of participants routinely consumed meat, poultry, fish, mopani worms, eggs and milk. A South African study suggested that because of the heightened risk and increased development of diabetes, the consumption of meat or animal products among patients with diabetes and their vulnerable FMs should be reduced.^30^ However, it is essential to consider other factors, such as affordability and cultural preferences, which may influence dietary choices. This well-established family dietary practice may contribute to worsening diabetes and elevate the risk of diabetes development in other FMs.

The study participants were well aware of the importance of exercise in controlling diabetes. However, it is crucial to note that knowledge alone does not necessarily translate into an active lifestyle, and patients may not be effectively encouraged to exercise.^14^ Factors such as urbanisation and other variables have been shown to impact physical activity levels, especially in rural areas.^31^ Exercise has been consistently linked to better diabetes outcomes for patients^32^ and a reduced risk of developing diabetes in at-risk populations.^33^ While most participants understood the advantages of patients monitoring their blood sugar levels before and after exercise, they lacked awareness of the specific exercise recommendations and their relationship with glucose readings. Because of their awareness of the benefits of exercise, a significant majority of survey respondents (87%) claimed to engage in regular exercise.

However, concerns arise when only 4.5% reported exercising in the week prior, suggesting inconsistency in their exercise routines. To promote consistent and sustainable exercise habits, behavioural modification techniques may be necessary to reinforce exercise motivation.

Adequate knowledge about diabetes management among FMs is linked to the adoption of appropriate practices. This includes making healthier food choices and increasing physical activity, driven by an understanding of the importance of a balanced diet and regular exercise. The practices adopted by FMs directly affect the health outcomes of individuals with diabetes.^34^ Proper practices, such as meal planning, portion control and exercise, can help in glycemic control, weight management and overall well-being.

Conversely, poor practices can lead to uncontrolled blood sugar levels, obesity and related complications among patients cared for by FMs. While knowledge and practices are critical, they do not exist in isolation. Upstream factors like access to healthcare, socio-economic status and cultural beliefs can influence both knowledge acquisition and the ability to implement practices.^10^ For instance, limited access to healthcare may result in inadequate diabetes education. Diabetes care and management also interact with downstream factors. These include complications arising from diabetes, such as neuropathy, nephropathy and retinopathy.^35^ These complications can, in turn, affect a family’s ability to provide care and support. The family system is central to diabetes care. Supportive family environments can facilitate adherence to recommended practices and positively impact DM outcomes.^36^

Conversely, family dynamics that hinder proper care can contribute to poor outcomes.

To address these issues, it is imperative to enhance FMs’ knowledge and practices through the adoption of a family-centred care approach. This strategy enables FMs of patients to collaborate and actively participate in their care. Educating FMs about proper diabetes management, including the do’s and don’ts, can lead to improved outcomes and a reduced prevalence of diabetes within families. Furthermore, the study recommends the implementation of local or community-based diabetes initiatives to increase public awareness and motivate individuals to adopt healthier lifestyles to prevent illness. These initiatives can play a vital role in promoting exercise and other healthy behaviours within the community. A multidisciplinary team is essential for providing comprehensive diabetes care, involving nutrition and exercise. General practitioners diagnose and manage diabetes, while registered dieticians create personalised meal plans and educate patients about healthy eating habits.

Certified diabetes educators help patients understand the disease, help in self-management, blood glucose monitoring and medication management. Physiotherapists design exercise programs, while psychologists address emotional challenges. Nurses assist in medication management, blood glucose monitoring and wound care. Pharmacists ensure proper medication adherence and monitor drug interactions. Social workers assess patients’ social and financial situations and connect them with community resources. Community health workers bridge the gap between healthcare providers and patients. This team approach ensures holistic care for diabetes, involving patients and families.

## Conclusion

Only few FMs had outstanding nutrition and exercise knowledge in diabetes management. However, these individuals also exhibit poor exercise and nutrition habits related to diabetic care, suggesting a potential risk of developing diabetes themselves. This limitation implies that their ability to provide effective support to their diabetic FMs is compromised.

To address this issue, there is a clear need to enhance family diabetes self-management education, with a specific focus on key aspects such as weight management, the importance of breakfast, increased intake of fruits and vegetables, exercise regimens and regular blood glucose monitoring. It is recommended to implement a comprehensive approach that combines family-oriented strategies, community-based awareness campaigns and behaviour change interventions. By taking these measures, the harmful effects on patients’ diabetes treatment can be minimised and also reduce the risk of FMs developing diabetes themselves.

### Recommendations

It is important to develop community-appropriate interventions, both in terms of diet and exercise interventions. It is also important to incorporate the opinions of FMs to know how to design effective interventions. People are far more inclined to participate in activities toward which they have contributed ideas.

### Limitations

This study did not evaluate the quality of support provided to patients. It also did not investigate whether FMs’ knowledge and skill levels were associated with how effectively patients were managing their diabetes. Association of knowledge levels and practice of FMs was not determined. Moreover, the study did not also include attitudes of FMs.
