# Corrigendum: Diet and exercise knowledge and practices for diabetes care within families in Senwabarwana

**DOI:** 10.4102/safp.v66i1.5921

**Published:** 2024-05-13

**Authors:** Mabitsela H. Mphasha, Linda Skaal, Tebogo Mothiba

**Affiliations:** 1 Department of Public Health, Faculty of Healthcare Sciences, University of Limpopo, Polokwane, South Africa; 2 Department of Research, Faculty of Healthcare Sciences, Durban University of Technology, Durban, South Africa; 3 Faculty of Healthcare Sciences, University of Limpopo, Polokwane, South Africa

In the original article published, Mphasha MH, Skaal L, Mothibal T. Diet and exercise knowledge and practices for diabetes care within families in Senwabarwana. S Afr Fam Pract. 2024;66(1), a5767. https://doi.org/10.4102/safp.v66i1.5767, one author’s last name was incorrectly spelt.

Instead of:

Tebogo Mothibal

It should be:

Tebogo Mothiba

The authors apologise for this error. The correction does not change the significance of study’s findings or overall interpretation of its results or the scientific conclusions of the article in any way.

